# Three-Dimensional Explant Platform for Studies on Choroid Plexus Epithelium

**DOI:** 10.3389/fncel.2020.00108

**Published:** 2020-05-05

**Authors:** Natalia Petersen, Lola Torz, Kristian H. Reveles Jensen, Gertrud Malene Hjortø, Katja Spiess, Mette Marie Rosenkilde

**Affiliations:** ^1^Novo Nordisk Foundation Center for Basic Metabolic Research, Faculty of Health and Medical Sciences, University of Copenhagen, Copenhagen, Denmark; ^2^Laboratory for Molecular Pharmacology, Department of Biomedical Sciences, Faculty of Health and Medical Sciences, University of Copenhagen, Copenhagen, Denmark

**Keywords:** *in vitro* model, tight junction markers, immune cells, choroid plexus, epithelium, organoid, explant, blood-cerebrospinal fluid barrier

## Abstract

The choroid plexus (CP) plays a major role in controlling the entry of substances and immune cells into the brain as it forms the blood-cerebrospinal fluid barrier (BCSFB) in the brain ventricles. Dysregulated immune cell trafficking through the epithelial cell (EC) layer of CP is central for the pathogenesis of infectious diseases in the brain and many neurodegenerative disorders. *In vitro* studies elucidating the function of the CP have so far been limited to the monolayer culture of CP ECs. To mimic immune cell migration across the CP barrier, a three-dimensional model would be advantageous. Here, we present an *in vitro* platform for studies of the immune cell trafficking based on CP explants/organoids. The explants were generated from fragments of mouse CPs in Matrigel, where the cells formed luminal spaces and could be maintained in culture for at least 8 weeks. We demonstrate expression of the major CP markers in the explants, including transthyretin and aquaporin 1 as well as ZO1 and ICAM-1, indicating a capacity for secretion of cerebrospinal fluid (CSF) and presence of tight junctions. CP explants displayed CP-like cell polarization and formed an intact EC barrier. We also show that the expression of transthyretin, transferrin, occludin and other genes associated with various functions of CP was maintained in the explants at similar levels as in native CP. By using dendritic cells and neutrophils, we show that the migration activity of immune cells and their interactions with CP epithelium can be monitored by microscopy. Thereby, the three-dimensional CP explant model can be used to study the cellular and molecular mechanisms mediating immune cell migration through CP epithelium and other functions of choroid EC. We propose this platform can potentially be used in the search for therapeutic targets and intervention strategies to improve control of (drug) substances and (immune) cell entry into the central nervous system.

## Introduction

The choroid plexus (CP), located in each of the brain ventricles, produces the cerebrospinal fluid (CSF), controls the access of substances and immune cells from the blood to the CSF and transfers substances from the CSF to the blood. Together with the arachnoid membrane, it forms the blood-cerebrospinal fluid barrier (BCSFB). Transthyretin (TTR) and transferrin play key roles in the production of CSF by the CP epithelium (Stauder et al., 1986; Strazielle and Ghersi-Egea, 2000). The CP is composed of a monolayer of specialized epithelial cells (ECs), enveloping capillaries formed by fenestrated endothelial cells. The capillaries in the CP allow free movement of molecules across the endothelial cell layer through fenestrations and intercellular gaps, which differs from the non-fenestrated continuous endothelium found elsewhere in the brain, where only smaller molecules such as water and ions are allowed to pass through intercellular clefts. The tight and adherent junctions of the CP epithelium are essential for the blood-CSF barrier function and the tightness of the epithelium determines the passive and active transport of molecules. The tight junctions are characterized by expression of zona occludens (ZO1), claudin proteins, ICAM-1 and VCAM (Bauer et al., 2014) and are central for the controlled passage of blood cells and immune cells across the BCSFB (Anderson and Van Itallie, 1995; Engelhardt and Sorokin, 2009). For selected immune cells, such as macrophages and dendritic cells (DCs), the CP allows regular crossing of the barrier as part of normal immune surveillance (Meeker et al., 2012). This process is restricted and tightly regulated. For many infectious diseases in the brain, pathogen entry through CP has been suggested, however, the mechanisms are not clear, partly due to the inability to study such interactions *in vitro* (Ghersi-Egea et al., 2018). It has been suggested that immune cells performing surveillance in the CP may directly be involved in viral infections and autoimmune processes (Meeker et al., 2012). Recent advances in understanding immune functions of CP call for a more sophisticated BCSFB model to investigate structure, function and barrier properties of the epithelial layer of CP. Several *in vitro* models have been invented and used for studies on CP structure and function—primary CP fragments (Inoue et al., 2015), monolayers (Baehr et al., 2006; Monnot and Zheng, 2013) and clusters (Sandrof et al., 2017). However, in these systems, the functions of CP cannot be easily monitored, as the functionality of the tissue depends on the ability to maintain 3D structure (Redzic, 2013; Takebe and Wells, 2019), i.e., appropriate cell polarization allowing formation of the lumen and thereby intact EC barrier. Cerebral organoids (Lancaster and Knoblich, 2015; Watanabe et al., 2017) are a recently developed advanced system, where induced pluripotent stem cells (iPSC) from human skin, are differentiated into a brain-like structure by “guidance” with specific growth factors. However, these cultures are not easily applied for studies using transgenic mouse material and individual mouse tissue comparison, as well as close monitoring of CP epithelium.

Here, we report a novel method for generation of dissociated CP explants, or CP fragment-derived organoid, from mice with essential morphology and characteristics of BCSFB, which we validate by immunostainings for CP markers, qPCR analysis and by monitoring interactions of immune cells with CP explants.

## Materials and Methods

### Animals

C57BL/6J mice were purchased from Charles River. Explants were generated from four to six mice per isolation. All animal experiments were approved by the Danish Animal Inspectorate (2018-15-0201-01442).

### Explant Generation

**Figure 1:**
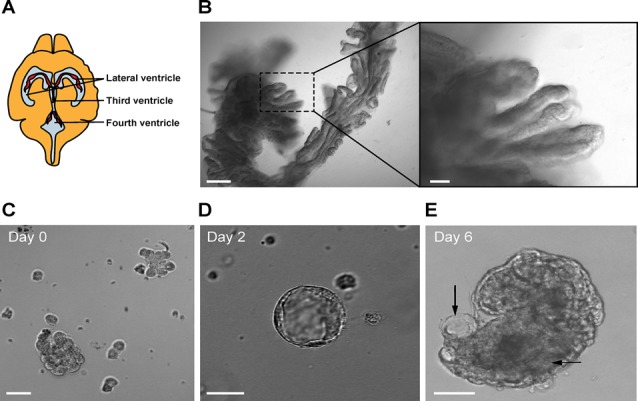
**(A)** Location of the choroid plexus (CP) in the ventricles of the mouse brain. **(B)** Mouse CP excised from the lateral ventricles (bar 100 μm). The right panel shows a magnified view (bar 20 μm). **(C)** Fragments of CP after trypsin digestion. Representative image of a primary CP cell cluster in Matrigel at day 0 (bar 50 μm). **(D)** Example of a small CP explant at day 2 (bar 20 μm). **(E)** Example of a CP explant at day 6 (bar 20 μm). Arrows show luminal spaces.

Non-fasted 8–10-week-old mice were euthanized by cervical dislocation, the skin on the back of the head and neck was sterilized with 70% ethanol and the brain was exposed using scissors, bone cutter, and fine forceps. The brain was excised and immersed in 10 ml of artificial CSF (aCSF) for 10 min to wash off the blood excess. The composition of aCSF was the following: 120 mM NaCl, 2.5 mM KCl, 1 mM NaH_2_PO_4_, 1.3 mM MgSO_4_, 17 mM Na-Hepes, 2, 5 mM CaCl_2_ and 10 mM glucose. The pH was adjusted to 7.4 with HCl. The CP from both lateral, third and fourth ventricles was dissected under a light microscope and immersed in 0.5 ml of aCSF on ice (Figure 1A). The procedure was repeated with the remaining three to five mice and the CP tissues were pooled in the 0.5 ml of the aCSF (Figure 1B). When all the tissues were collected, they were minced with a pair of fine ophthalmologic scissors for 5 min to produce roughly 1-mm cubes. After mincing, the total volume was brought up to 1 ml by the addition of 0.5 ml aCSF. The tube contents were briefly spun and the supernatant was removed.

### Tissue Digestion

**Figure 2:**
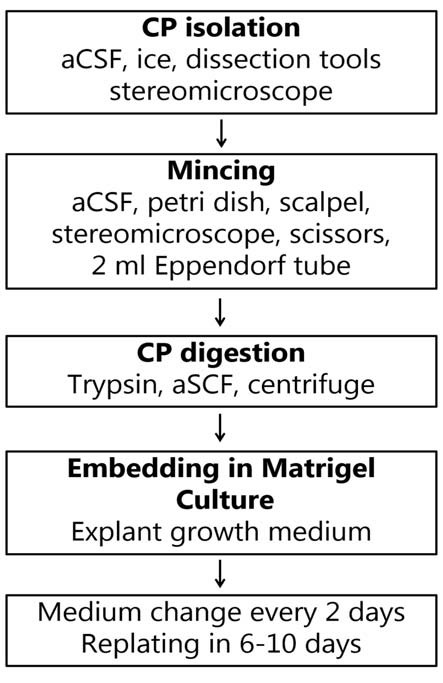
Flow chart for the generation of CP explants.

Digestion solution contained 0.25% trypsin (Life Technologies) in aCSF, and 1 mol/l EDTA (Life Technologies™), freshly prepared and kept on ice until use. One milliliter of digestion solution was added to the tube’s contents and mixed by gently tapping the tube. The supernatant was aspirated using a pipette and 1–2 ml of digestion solution was added. The tube was incubated at 37°C for 15–20 min with gentle pipetting with a plastic Pasteur pipette every 5 min. After the digestion, the tissue was further dispersed by gentle pipetting and checked under a microscope for the presence of small fragments of CP (Figure 1C). The digestion was stopped by adding 4 ml of DMEM containing 5% fetal bovine serum (FBS) to the digestion mixture and filtered through a 70 μm filter. The tube was centrifuged at 300× *g* for 5 min at 4°C in a 1.5 ml sterile tube. The supernatant was discarded and the pellet was washed once more with DMEM containing 5% FBS by resuspension and centrifuged again. At this point, the pellet contained clusters of primary EC ranging from 20 to 50 μm. The pellet was resuspended in 200 μl of growth medium (the amount is given per pooled four CPs), containing DMEM with 5% FBS, supplemented with HEPES, L-glutamine (Sigma) and primocin 1:500 (Thermo Fisher Scientific), 1 μg/ml EGF (Peprotech), 1 μg/ml FGF10 (Peprotech), 1 μg/ml IGF1 (Peprotech), and 1:200 B27 (Life Technologies). A 20 μl aliquot of cell suspension was mixed with 20 μl of 0.4% trypan blue to count cell numbers and to assess the viability. The ice-cold contents of the tube were mixed with 200 μl of Matrigel (growth factor reduced, from Corning) and 20–50 μl of the suspension was distributed in the center in each well of a 24-well glass-bottomed plated (Corning). The growth medium was added to the wells after the Matrigel domes solidified. The plate was then placed in a humidified incubator with 95% air/5% CO_2_ at 37°C. The growth medium was used during the first 6 days of culture. Every two days, half of the medium volume was replaced with fresh growth medium. On the second day, 20 μM cytosine arabinoside (Ara-C) was added to the culture medium. After 6 days, the explants were re-plated in fresh Matrigel to eliminate cell debris and blood vessels, and to further dissociate large tissue fragments to allow explant formation. The explants were released from Matrigel by pipetting and embedded in fresh Matrigel every following 6–10 days. Ara-C treatment was applied in every passage for 48 h to maintain fibroblast-free cultures. After the re-plating (passaging) the cultures were maintained in the growth medium, but three days before the experiments, FBS was omitted from the medium composition to promote cell maturation (Hakvoort et al., 1998; Barkho and Monuki, 2015). We maintained the cultures for at least 8 weeks. The procedure for explant generation is presented in Figure 2.

### Staining of Explants in Whole-Mount Preparations

For immunostainings, the medium was removed and explants were first fixed in 0.5 ml of 4% PFA in each well at 4°C for 30 min. After that, the explants were washed with PBS at room temperature during 10 min three times and treated with the permeabilization buffer containing 0.15% Triton X-100 in PBS. Next, the samples were blocked with 2% BSA for 60 min and incubated with the primary antibodies overnight at 4°C. Anti-transthyretin (anti-TTR; Santa Cruz) was used at 1:500, anti-aquaporin 1 (AQ-1) and anti-ZO1 (both from Abcam) were used at 1:200 dilution. After washing three times with PBS (10 min per wash), the samples were incubated with the secondary antibody Alexa Fluor 488 diluted 1:500 and DAPI for 1 h at 37°C. After washing with PBS, the samples were examined using confocal microscopy. Fragments of isolated CP fixed in 4% PFA were used as a positive control. Phalloidin Alexa Fluor 647 was applied in fixed wholemount preparations according to the manufacturer’s instructions for 15 min and washed with PBS before viewing. Images were acquired on a confocal Leica 780 microscope and processed using Zen software and ImageJ. Immunostainings were performed on two wells for each antibody on two batches of explants derived from material from three to five mice.

### RT-PCR Analysis

Explants were washed with cold PBS and collected from wells together with Matrigel, transferred to a 1.5 ml conical bottomed tube and pipetted to disrupt Matrigel. Then the tube was briefly spanned and the supernatant was removed. Total RNA was extracted from the explants or isolated CP (as a positive control) using the RNeasy Micro Kit (Qiagen) according to the manufacturer’s instructions. The cDNA synthesis was performed on 100 ng of total RNA, using Superscript III reverse transcriptase with oligo dT primers according to the manufacturer’s instructions (Invitrogen, Thermo Fisher Scientific). Quantitative real-time PCR was performed using the QuantStudio 6 Flex Real-Time PCR System (Thermo Fisher Scientific) and SYBR green assays with primers at 10 nM was used according to manufacturer’s instructions. Each gene expression was tested in three explant lines (biological replicates) generated from nine mice in independent experiments. The individual measurements were averaged from triplicated qPCR wells (technical replicates). Cycle threshold (Ct) values were obtained using the QuantStudio Real-time PCR Software, and the Δ−ΔCt method was used to calculate the relative fold change of cDNA levels compared to the reference gene (*Ywhaz*). Primer sequences are presented in Table 1.

**Table 1:** Quantitative transcription-polymerase chain reaction (RT-PCR) mouse primers.

Gene	Alias	Forward	Reverse
Tyrosine 3-Monooxygenase/Tryptophan 5-Monooxygenase	Ywhaz	AGA CGG AAG GTG CTG AGA AA	GAA GCA TTG GGG ATC AAG AA
Transthyretin	Ttr	AGCCCTTTGCCTCTGGGAAGAC	TGCGATGGTGTAGTGGCGATGG
Matrix metallopeptidase 9	Mmp9	AAGGACGGCCTTCTGGCACACGCCTTT	GTGGTATAGTGGGACACATAGTGG
Prostaglandin D2 synthase (brain)	Ptdgs	GGGAATCCCAAGAGACCCAG	GCTCTGAGCAAATGGCTGC
Occludin	Ocln	ATGACTTCAGGCAGCCTCGTTACA	TCAGCAGCAGCCATGTACTCTTCA
Transferrin	Tf	GGAAGACTCTGCTTTGCAGCTAT	GCCCAGGTAGCCACTCATGA
Creatine kinase brain	Ckb	GGAATCCTCACCTGGGCT	GGCAGGCCAAACCCTAGT
Insulin-like growth factor	IGF-2	GTCGATGTTGGTGCTTCTCA	AAGCAGCACTCTTCCACGAT
G protein-coupled receptor 125	GPR125	TTT CTG ACT ATG GGC GAA GG	GCC TCC ATG ATG GTG TTC TT

*Related to methods*.

### Immune Cell Migration Studies

**Figure 3:**
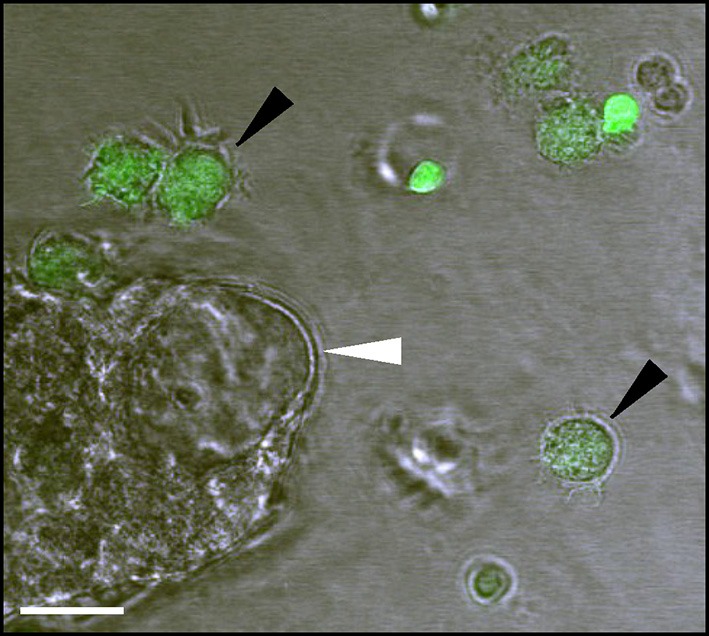
Experimental design for testing the immune cell activity towards the explants (white arrow). Dendritic cells (DCs) are labeled green with fluorescent marker carboxyfluorescein succinimidyl ester (CFSE; black arrows; bar 20 μm).

For a demonstration of immune cell interaction with CP explants, we tested two immune cell types: Mouse neutrophils (MN) and human DCs. DCs were obtained from human blood as described previously (Jørgensen et al., 2018). In brief, DCs were prepared from human peripheral blood mononuclear cells (PBMCs) isolated from buffy coats (obtained from healthy donors), by centrifugation on a Lymphoprep gradient (STEMCELL technologies). Monocytes were isolated from PBMC by plastic adherence. Adhered monocytes were subsequently cultured and differentiated into immature DCs by incubation with IL-4 (250 U/ml) and GM-CSF (1,000 U/ml) for 6 days, followed by activation into mature DCs by incubation with 1,000 U/ml IL-6, 1,000 U/ml IL-1β, 1,000 U/ml TNF-α (all from Peprotech), and 1 μg/ml PGE2 (from Sigma) for an additional 2 days in the same medium. Before the experiment, frozen DC was thawed in a water bath at 37°C and washed with warm X-Vivo 15 medium (Lonza) supplemented with 2% human AB serum and glutamine (both from Sigma), spun down and resuspended in fresh medium to remove DMSO. MN were prepared from 400 μl of peripheral mouse blood, by adding 10 ml of distilled water, quickly mixing and adding 2X concentrated HBSS supplemented with 10% FBS. The following purification was done as described earlier (Swamydas et al., 2015), using density gradient centrifugation with Histopaque 1077 and 1119 (Sigma). DCs and MNs were then labeled with 1 μM fluorescent marker carboxyfluorescein succinimidyl ester (CFSE; Cell Labeling Kit, Abcam) in the experiment medium during 5 min and washed with fresh medium. Explant and immune cell suspension (20,000 cells per well) were mixed into an aliquot of Matrigel (1:1) together with a combination of human interleukin 6, interleukin 1 and TNFα (all at 1,000 U/ml) and distributed in a 48-well glass-bottom plate as 20 μl drops. The plated material was allowed to solidify at 37° C for 5 min before the experiment medium was added. The plate was transferred to a confocal microscope for monitoring. The bright field and fluorescence images at 488 nm throughout the explant volume were taken every 2 min during the first 4 h. Alternatively, a time-lapse was recorded during 24 h in a wide-field microscope and the explants were examined after the time-lapse under a confocal microscope to evaluate the number of transmigrating cells. The immune cells in similar experiments have been shown to display activity for 48 h in our earlier studies (Sebrell et al., 2019). Each explant was imaged as a z-stack and every plane was examined to ensure the location of immune cells. A representative image of the experimental setup showing CP explant and interaction with fluorescence-labeled immune cells is presented in Figure 3. The explants were loaded with CFSE as described for DC labeling, washed, incubated with fresh medium for 30 min and monitored in a microscope for 3 h. The mean fluorescence intensity was measured over the lumen area on the images using ImageJ. Organoid images were acquired on a confocal microscope with an optical slice thickness of 3 μm with 3 μm between the sections. The measurements were performed on the section with the largest organoid diameter over the 3 h of the experiment. We did not observe significant changed in the lumen area during the observation period. The acquisition settings, such as exposure time, laser intensity and gain, were not changed during the experiment.

### Statistics

Quantitative results are presented as mean values with standard error of the mean (SEM). A comparison of two groups was performed by the Wilcoxon rank-sum test. For statistics, we used R: A language and environment for statistical computing. R Foundation for Statistical Computing, Vienna, Austria^1^. qPCR data were analyzed using Wilcoxon matched-pairs signed rank-test to compare the data from different explant lines. *P*-values less than 0.05 were considered significant (*< 0.05; **< 0.01; *** < 0.001).

## Results

### CP Explants Display Markers of CP Epithelial Cells

**Figure 4:**
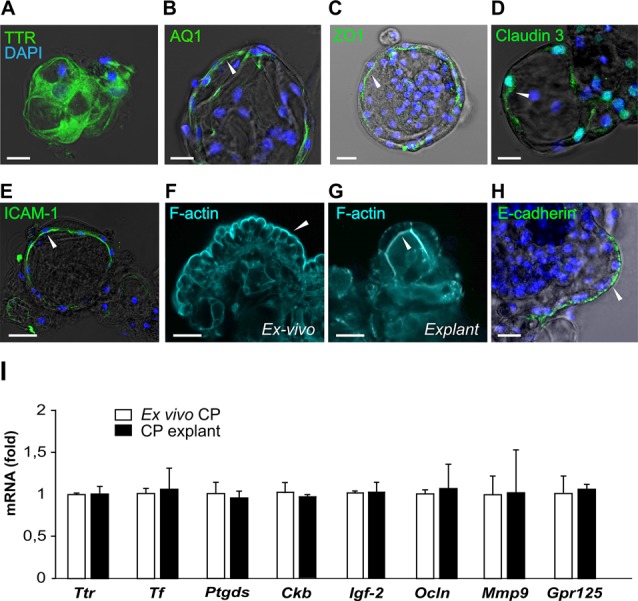
CP explants show typical CP markers. **(A–E)** Presence of transthyretin (TTR), aquaporin 1 (AQ1), ZO1, Claudin 3 and ICAM-1 with DAPI (blue) staining in CP explants. White arrows indicate the localization of the staining on the apical or basolateral cell membrane (bar 20 μm). Optical thickness of images sections 3 μm. **(F)** Distribution of F-actin (cyan) detected by phalloidin in *ex vivo* CP fragment and explants **(G)**. **(H)** E-cadherin staining in a CP explant. **(I)** Expression of the CP epithelial markers, *Ttr*, lipocalin-type prostaglandin D Synthase (*Ptgds*), transferrin (*Tf*), creatine kinase brain (*Ckb*), occludin (*Ocln*), insulin-like growth factor 2 (*IGF-2*) and G protein-coupled receptor 125 (GPR125) in native mouse CP (white bars) and CP explant at day 12 (black bars). *n* = 3 for explant series and *n* = 4 for CP fragments.

CP cell clusters formed cyst-like structures starting from day 2 and onward (Figures 1C–E), following typical organoid formation (Dahl-Jensen and Grapin-Botton, 2017). Explants were formed with high efficiency as nearly all fragments developed cystic structures and could be maintained for at least 8 weeks with re-plating in fresh Matrigel every 6–10 days. The explants had one or several well-defined cavities or “lumens” (Figures 1D,E). The explant size at this point was ranging from approximately 50 to 400 μm, depending on the initial digestion efficiency. The explant shape varied significantly, as explants were forming multiple lumens of smaller size or one lumen of larger size. We have not observed major differences in the explant size or shape between the batches. Immunostaining for specific choroid epithelium markers TTR, AQ-1, ZO1 (zonula occludens) and claudin 3 (Stauder et al., 1986; Ghersi-Egea et al., 2018), showed positive labeling in all cells constituting explants (Figures 4A–D). We performed immunostaining for ICAM-1 as one of the major and specific CP markers of tight junctions (Steffen et al., 1996). Although many cells appeared flattened, possibly due to quick filling up of the lumen with CSF, they were polarized and demonstrated positive immunostaining for ICAM-1 on the apical cell membranes (Figure 4E). F-actin filaments are associated with tight junctions on the apical surface and directly involved in controlling paracellular permeability (Anderson and Van Itallie, 1995). To visualize F-actin distribution in explants, we used phalloidin 677 (Invitrogen). As Figures 4F,G show, the staining was more pronounced on the inner surface of the explants (Figure 4G), than on the outer surface, while in primary CP tissue it was mostly localized on the outer surface of fragments (Figure 4F; facing the ventricle lumen *in vivo*). This confirmed that the apical side was facing the explant lumen. The main structural differences and similarities of native CP tissue and CP-fragment derived explants are schematically presented in Figure 7. Similar cell polarization has been observed in other types of organoids, mimicking hollow organs, such as intestinal organoids (Fatehullah et al., 2013; Petersen et al., 2018). Consistent with this, E-cadherin labeling was localized to the basolateral side (Figure 4H). Thus, CP explants were capable of self-organizing into polarized cell layers and forming their lumen with an intact epithelial barrier, the presence of which is supported by the retention of fluorescent dye CFSE inside of explants (Supplementary Figures S1A,B).

**Figure 5:**
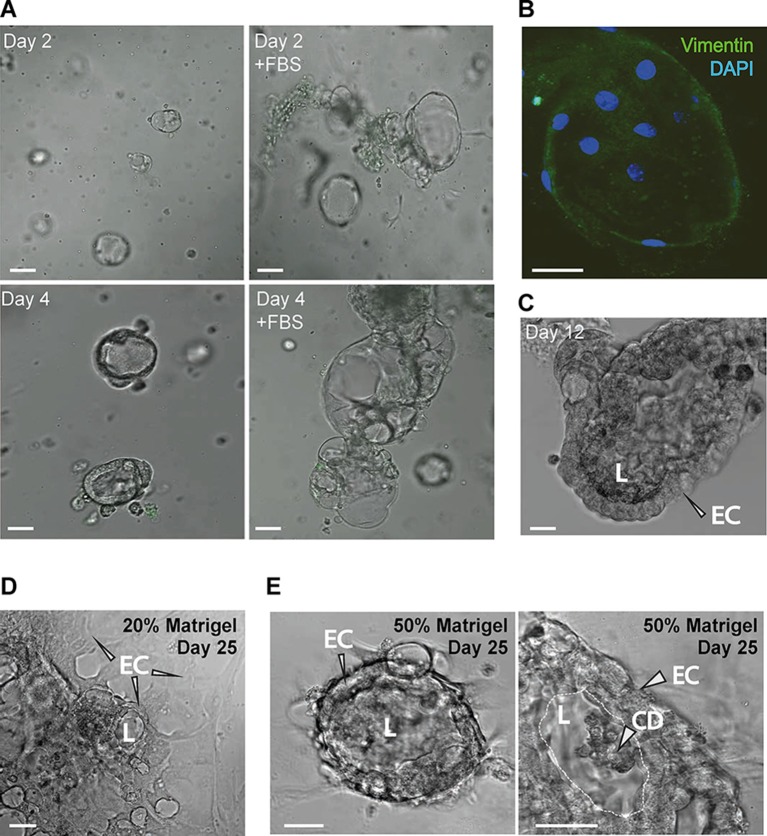
**(A)** The addition of FBS increases the CP explant growth (bar 20 μm). **(B)** Ara-C-treated CP explants show very few fibroblasts labeled by vimentin immunostaining (bar 10 μm). **(C)** CP ex cultured for 12 days. FBS was withdrawn during the last 3 days. **(D,E)** CP explants plated in 20% and 50% Matrigel on day 25. Panel to the right shows a magnified view of the epithelial cells (ECs), lumen (L) and accumulation of cell debris (CD) in the lumen (bar 20 μm).

**Figure 6:**
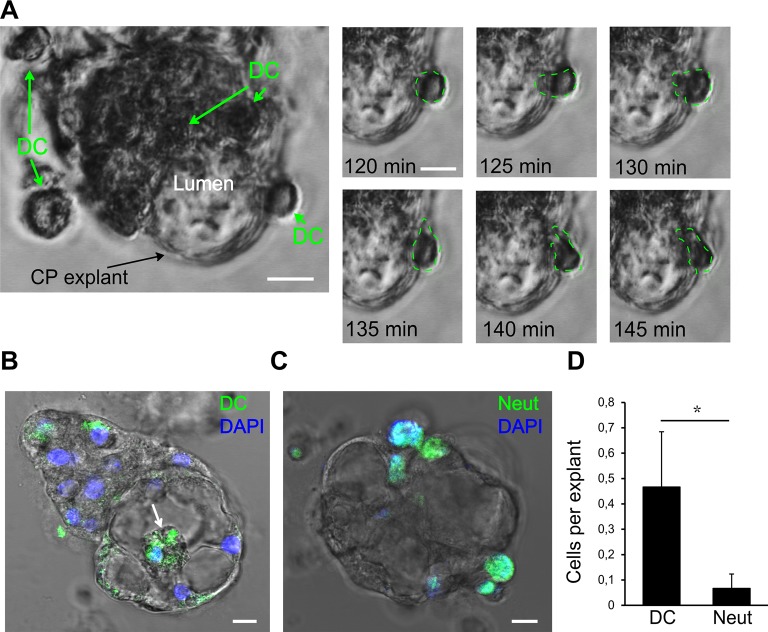
Interaction of human dendritic cells (DCs) with CP epithelium. **(A)** A representative migration experiment with human DCs interacting with the CP explant (bar 10 μm). Time series shows one of the DCs crossing into the lumen space. The last 35 min of the total of 100 min of the experiment are shown. **(B)** Human DCs labeled with CFSE (green) migrate into the lumen (white arrow). Endpoint image after the experiment. **(C)** Mouse neutrophils (MN) labeled with CFSE (Neut, green arrows) attached to CP explant. **(D)** Comparison of DC and neutrophil (Neut) transmigration into the explant lumen during 24 h. Data are means ± SEM. *p < 0.05 by Wilcoxon rank-sum test, *n* = 20.

**Figure 7:**
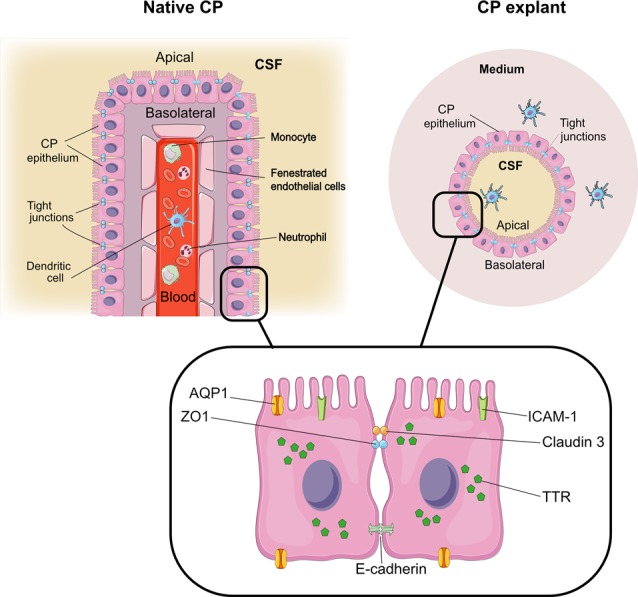
CP explants as a model to study interactions with immune cells. Comparison of architecture in native CP and CP explants. The graphical elements were modified from SMART SERVIER MEDICAL ART, Les Laboratoires Sevier.

The expression of key CP markers determined by reverse transcription-polymerase chain reaction (RT-PCR) was compared in the explants from passage 2 (day 12) and native CP tissues. We chose markers previously identified as highly expressed in CP (Marques et al., 2011): *Ttr* and transferrin (*Tf*), as well as the major protein of the CSF, lipocalin-type prostaglandin D Synthase (*Ptgds*), the creatine kinase brain type (Ckb), important for creatine phosphorylation, and insulin-like growth factor 2 (IGF-2; Péraldi-Roux et al., 1990; Blödorn et al., 1996; Marques et al., 2011; Lun et al., 2015). The expression of occludin (Ocln) and matrix metallopeptidase 9 (Mmp9), important for the maintenance of tight junctions (Martins et al., 2011), was also determined as well as the expression of GPR125, an adhesion G protein-coupled receptor highly expressed in CP EC (Pickering et al., 2008) and recently shown to be constitutively internalized (Spiess et al., 2019). Our data show that these markers were expressed in explants and *ex vivo* CP at similar levels (Figure 4I), indicating that the CP EC maintained their identity in the applied culture conditions.

### Improved CP Explant Culture Efficiency With Serum

To maximize the efficiency of CP explant cultures, we used FBS to stimulate proliferation and cytosine arabinoside (Ara-C) to avoid fibroblast proliferation. Addition of FBS showed a pronounced positive effect on cell growth (Figure 5A), in agreement with published work (Barkho Barkho and Monuki, 2015). While, Ara-C treatment resulted in few immunopositive cells, determined by anti-vimentin staining, indicating low numbers of mesenchymal cells in explant cultures (Figure 5B). After 9 days of culture with FBS, the culture medium was changed to a medium without FBS, after 3 days the explant maintained its original structure (Figure 5C), indicating the importance of FBS for the early establishment of the explants. Using the same culture medium combination, we tested lower Matrigel content (20%) in plating suspension and found that explants were also able to adapt to two-dimensional culture while still forming “lumens” (Figure 5D). After prolonged culture, explants in 50% Matrigel increased in size and the cells assumed nearly columnar morphology at day 12–18 and started to accumulated CD in the lumen (Figure 5E).

### Monitoring Immune Cell Interactions in CP Explants

Next, we investigated the possibility of monitoring the interactions of immune cells with CP explants. DCs are professional effective antigen-presenting cells and play important roles in immune surveillance of the brain together with macrophages (McMenamin, 1999; Serot et al., 2000; Meeker et al., 2012). Under normal circumstances, macrophages and DCs migrate through the CP epithelial layer for immune surveillance purposes, but these cells can also harbor infections, such as pathogenic viruses (Meeker et al., 2012; Quintana et al., 2015). DC transmigration is thought to play a particular role in the pathogenesis of multiple sclerosis (Vercellino et al., 2008; De Laere et al., 2018). Under pathological conditions, such as brain injuries and infections, other immune cells, such as neutrophils, cross through the CP and accumulate in the CSF (Szmydynger-Chodobska et al., 2009; Kaur et al., 2016). As an example of an interaction of immune cells with the CP epithelial layer, we tested two immunocompetent cell types—activated DCs and neutrophils, which have different capacities of traveling inside the CP (Kaur et al., 2016). We added human DCs and MN to CP explant cultures and monitored the immune cell migration in the presence of the cytokines: IL1, IL6, and TNFα. During the 24 h incubation, DCs migrated towards explant cells, and some of them migrated into the explant lumen (Figure 6A). To be able to quantify DCs crossing the epithelial barrier, we labeled the DCs with CFSE, a cell-permeable fluorescent cell staining dye, and visualized their location inside of the lumen space after 24 h (Figure 6B). MN also migrated towards the explants and made contact with CP cells but very few of them were able to penetrate and migrate inside (Figure 6D, Supplementary Movie S1). Wilcoxon rank-sum test with continuity correction resulted in a *p*-value = 0.04734. Considering the data and actual shift in the mean of the distribution, a power of the experiment can be simulated to be above 0.9 for the Wilcoxon rank-sum test using the wmwpow package in R. These data suggest that interactions of immune cells with CP epithelial border and their transmigration can be determined *in vitro* in the explant/organoid platform by microscopy.

## Discussion

Organoids as research platform is widely used for several epithelial tissues, such as intestine, liver, thyroid and mammary glands (Rossi et al., 2018). These systems mimic the structure of the epithelium *in situ* due to embedding in a gel, resembling an extracellular matrix (Takebe and Wells, 2019), which allows the three-dimensional tissue formation. Here, we report an *in vitro* platform for the generation of native CP fragment-derived organoids, or explants. The term “organoid” is often used with regards to stem cell generated cultures of native tissue explants, capable of showing functional and morphological features of an organ (Fatehullah et al., 2016). In this study, the CP explants/organoids were generated from dissociated fragments of adult mouse CP, could be passaged and could reorganize themselves and form a closed lumen. The explants can be generated from individual mice, which makes it advantageous compared to iPSC derived cerebral organoids. Indeed, iPSC-derived organoids tend to resemble fetal tissues and do not have the appropriate functionality of adult stem cell-derived organoids (Takebe and Wells, 2019). The CP explants mainly consist of one cell type, CP EC, without contamination of blood vessels and fibroblasts, which makes them a suitable model for gene expression studies. Furthermore, as practice shows, the generation of cerebral organoids relies on a long-term culture supplemented with expensive growth factors (Lancaster and Knoblich, 2015), while this CP explant method presented here is less expensive and time-consuming.

The three-dimensional culture conditions allow the cell lining to form lumen, which makes immune cell transmigration studies possible. Explants can be generated in various sizes, allowing the system to be adapted for different kinds of microscopy. These cultures can also be transformed into nearly monolayer cultures for wide-field imaging after the primary explants have been established. Thus, the flexibility of the system is another benefit of CP explants compared to other culture systems, such as CP epithelium monolayers and microspheres.

The choroidal explant cultures established from mouse CP in this study display proper morphology and cell polarization. Immunochemical studies demonstrated the presence of CP markers: Ttr, a thyroxine transport protein exclusively produced by the choroidal epithelia in the CNS, AQ-1, ZO1 and the tight junction marker ICAM-1. Gene expression of transthyretin and transferrin was maintained in explants at similar levels to native CP, which indicates the ability of explants to secret CSF products. Gene expression levels of *Ptgds*, which plays a role in immune cell recruitment and maintenance of extracellular matrix, were similar in CP explants and native CP. This is important with regards to future modeling of pathophysiological conditions in the explant system, such as multiple sclerosis. Expression of AQ1, a water channel, on the apical side, indicates the appropriate polarization of explant cells, supported by F-actin staining. However, future studies, such as analysis of produced and secreted proteins and appropriate expression of receptors, are needed to address whether the functions of secretion metabolic control and transport in CP explant cells are similar to those in native CP. The ability of CP explants to generate cyst-like lumens after plating in Matrigel and the presence of tight junction markers ICAM-1, ZO1 and occludin indicates that CP explant cells provide intact barrier function. The detection of epithelial barrier impairment in the explants has limitations when using a fluorescence dye in the lumen because it is difficult to estimate the changes in fluorescence intensity with high precision. Penetration of Evans Blue or dextran-based dyes into the CP explant lumen from the culture medium can be used to detect compromised epithelial barrier integrity. In this study, we present a proof of principle for monitoring the interaction of immune cells with CP cells. This opens a possibility to study the behavior of various immune cells during pathogenic processes, such as transport of viruses across the CP epithelial barrier or nerve tissue damage in, for example, multiple sclerosis by lymphocytes and monocytes migration across the barrier, a process mediated by cytokines and facilitated by adhesion molecules on the immune cells. Further studies are needed to address whether all types of immune cells display their “normal” behavior towards CP cells *in vitro*, as well as determine which proteins are secreted by the CP explants into the lumen, or how the cell membrane capacity is changing when cells are cultured *in vitro*. In relevant studies, the integrity of CP epithelial barrier and immune cell activity can be modified by the addition of, for example, LPS or cytokines and complement proteins. Besides, this system can potentially be used to test the drug’s ability to cross the barrier or to induce its opening, to model disease by increase inflammatory signaling.

The limitation of this system is the variability of explants in size, and that large single luminal spaces are challenging to generate and require several weeks to be formed. However, this can be overcome by changing the cell culture regimes, such as applying FBS and careful selection of additional growth factors, which have been proven to improve the culture of some organoid types (Fujii et al., 2018). Importantly, as with many organoid systems (Rossi et al., 2018; Takebe and Wells, 2019), the full identity of cells constituting CP is not determined, and they develop in the artificially composed environment, where they cannot benefit from systemic influences and factors produced by neighboring tissues. It remains to be determined to what extent the functions of EC resemble those *in vivo*. However, although CP explants do not represent a full model of BCSFB and cannot secrete full-fledged CSF, as they do not have functioning blood vessels, our results show that they can be used to test specific functions of CP epithelium, such as cell-to-cell connections, gene expression profiling and expression of proteins in different conditions. For example, in addition to the applications mentioned in this study, the CP explant model can be useful to study metabolic conditions and diseases, such as aging, ischemia, Alzheimer’s disease, Parkinson’s disease, and as already mentioned multiple sclerosis. These conditions have been associated with impairment of CP secretory capacity, amyloid clearance and barrier function, where the transport of pathogenic particles is facilitated by changing properties of the cell membrane, in addition to poorly controlled trafficking of immune cells (for review, Cheng and Haorah, 2019). Disease modeling using the CP explants can be applied for dissecting the mechanisms of such impairment and identification of regulatory components, such as structural proteins or receptors. In this model, gene and protein profiles can be analyzed and secreted products and growth factors can be detected in the medium. Particular metabolic parameters, such as labeling of mitochondria and their activity using voltage-sensitive dyes or markers of ATP synthesis (Kant et al., 2018), could be also accessed in the platform.

Thus, the CP explant model can be used to study the cellular and molecular mechanisms mediating immune cell migration through CP epithelium and other functions of CP. In perspective, studies on the regulation of migratory behavior of immune cells in CP can identify new therapeutic targets and develop strategies to interfere with the recruitment of pathogenic immune cells to the CNS.

## Data Availability Statement

The datasets generated for this study are available on request to the corresponding author.

## Ethics Statement

All experiments and procedures were approved by the Department of Experimental Medicine and performed according to procedures laid out by the Danish Ministry of Justice and the Danish National Committee for Ethics in Animal Research and guidelines of the Council of the European Union (86/609/EEC).

## Author Contributions

NP, LT, and KS designed the research study, conducted the experiments, analyzed the data, and wrote the manuscript. KJ and GH contributed to the experimental work, analyzed the data, and revised the manuscript. MR designed the research study, analyzed the data, and wrote the manuscript. All authors commented on and approved the final manuscript.

## Conflict of Interest

The authors declare that the research was conducted in the absence of any commercial or financial relationships that could be construed as a potential conflict of interest.

## Footnotes

^1^https://www.R-project.org/

## Supplementary Material

FIGURE S1 **(A)** Retention of cell-permeable fluorescent dye CFSE (green) in the lumen of a CP explant over time (bar 20 μm). **(B)** Mean fluorescence intensity over the lumen area from CFSE loaded organoids (*n* = 8). Representative traces from 8 organoids during the same imaging experiment as shown in panel **(A)**.

MOVIE S1 This movie shows transmitted light time-lapse images of two closely positioned mouse CP explants in Matrigel co-cultured with mouse neutrophils, some of which migrate towards the explants. One of the neutrophils repeatedly “investigates” the explant structures, but do not transmigrate inside. The time-lapse covers 2 h. A similar experimental setup is also illustrated in Figure 6C.

## Abbreviations

Ara-C: Cytosine arabinoside
BCSFB: Blood-cerebrospinal fluid barrier
CFSE: Carboxyfluorescein succinimidyl ester
CP: Choroid Plexus
CSF: cerebrospinal fluid
DCs: Dendritic cells
Neut: Mouse neutrophils
PBMCs: Peripheral blood mononuclear cells.
