# Supplementary figures and images for: Three-Dimensional Explant Platform for Studies on Choroid Plexus Epithelium
Source: Front Cell Neurosci. 2020 May 5;14:108. doi: 10.3389/fncel.2020.00108 (PMC7214744; doi:10.3389/fncel.2020.00108)

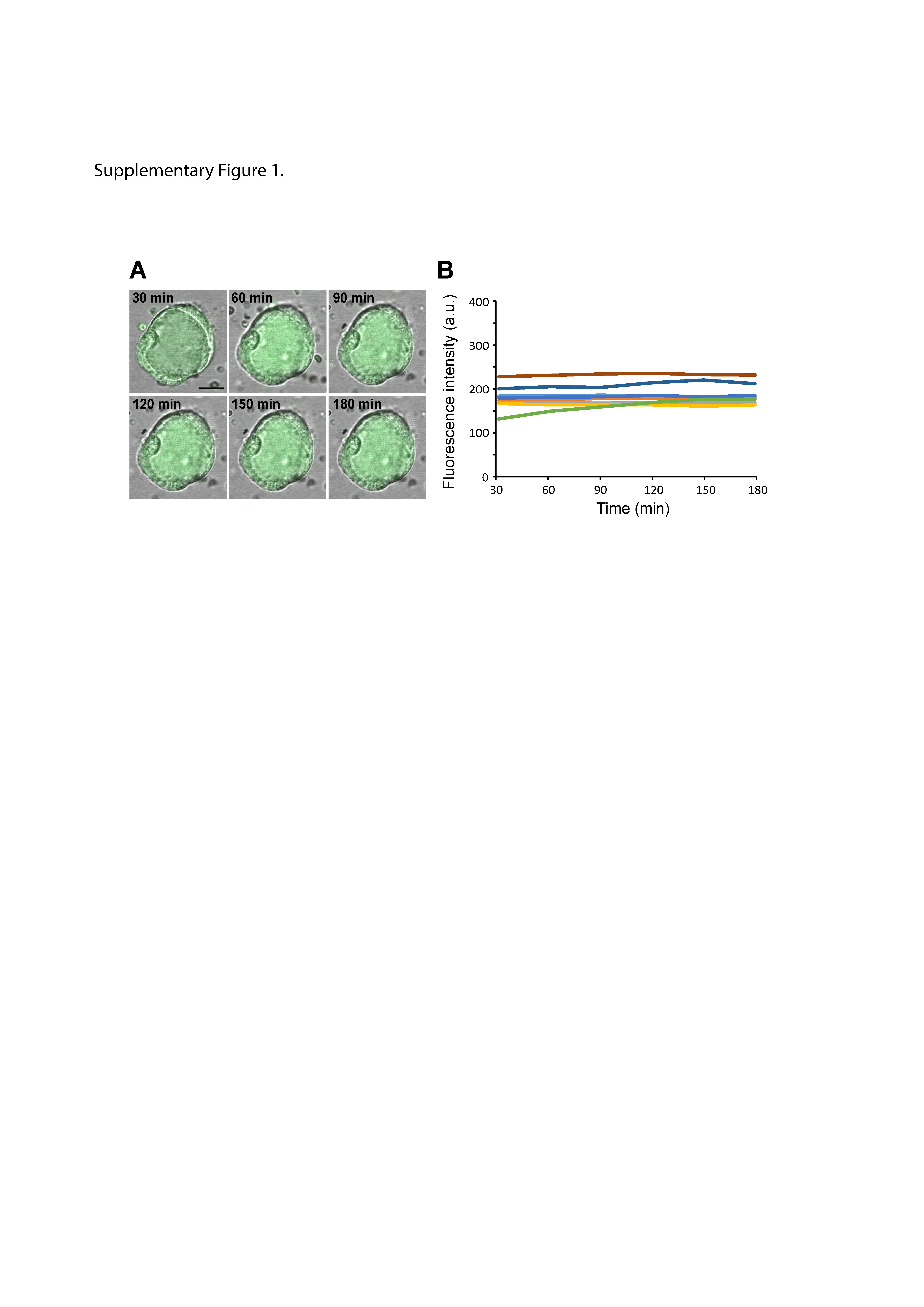

Supplement: FIGURE S1 — (A) Retention of cell-permeable fluorescent dye CFSE (green) in the lumen of a CP explant over time (bar 20 μm). (B) Mean fluorescence intensity over the lumen area from CFSE loaded organoids (n = 8). Representative traces from 8 organoids during the same imaging experiment as shown in panel (A). [file Image_1.TIFF]
